# Association Between Human Papillomavirus Vaccination and the Risk of Cervical Cancer and Precancerous Lesions in Israel: A Retrospective Cohort Study

**DOI:** 10.3390/jcm15030995

**Published:** 2026-01-26

**Authors:** Gabriel Chodick, Myriam Strassberg

**Affiliations:** School of Public Health, Gray Faculty of Medical & Health Sciences, Tel Aviv University, Tel Aviv 6997801, Israel; strassberg@mail.tau.ac.il

**Keywords:** human papillomavirus, HPV vaccine, cervical cancer, cervical intraepithelial neoplasia, cohort study, Israel

## Abstract

**Background**: Human papillomavirus (HPV) infection is the necessary cause of almost all cervical cancers. HPV vaccination programs have been implemented worldwide, yet real-world evidence on vaccine effectiveness against invasive cervical cancer remains limited. **Methods**: We conducted a retrospective cohort study using synthetically generated data from a large health provider in Israel, including women who underwent a first Papanicolaou (Pap) test during 2014–2015. Their HPV-vaccination status before an index Pap test was obtained from computerized records. Incident cervical cancer and high-grade cervical pathology (cervical cancer, cervical intraepithelial neoplasia [CIN] 1–3, and carcinoma in situ) occurrence were examined through 2022. Hazard ratios (HRs) and 95% confidence intervals (CIs) were estimated using Cox proportional hazards models and fitted with propensity score weighting. **Results**: The cohort included 98,102 women, of whom 9198 (9.4%) were vaccinated against HPV before an index Pap test. While HPV-vaccinated women had a higher risk of cervical pathology compared with unvaccinated women, among women vaccinated before age 18, HPV vaccination was associated with a substantially lower, though not statistically significant, risk of cervical cancer (HR 0.28, 95% CI: 0.07–1.20, *p* = 0.087). **Conclusions**: In this large cohort, HPV vaccination was correlated with a higher risk of cervical pathology, likely reflecting residual confounding factors from sexual behavior and differential baseline risks of HPV infection. In contrast, vaccination during adolescence showed a marked trend toward a reduced risk of cervical cancer, consistent with international evidence that early vaccination, prior to HPV exposure, is the most effective preventative treatment.

## 1. Introduction

Human papillomavirus (HPV) is the most common sexually transmitted infection worldwide and a major cause of morbidity and mortality in both women and men. High-risk HPV types, particularly HPV-16 and HPV-18, are responsible for the vast majority of cervical cancers and a substantial proportion of other anogenital and oropharyngeal malignancies. The global burden of HPV-related disease remains considerable, especially in settings with limited access to screening and vaccination [1].

HPV is a double-stranded DNA virus of the *Papillomaviridae* family. More than 200 HPV genotypes have been identified and are classified as low-risk or high-risk according to their oncogenic potential. Low-risk types (e.g., HPV-6 and HPV-11) commonly cause benign anogenital warts, whereas high-risk types, especially HPV-16 and HPV-18, are etiologically linked to cervical intraepithelial neoplasia (CIN) and cervical cancer, as well as cancers of the vulva, vagina, penis, and anus, as well as a subset of head and neck cancers [2]. A persistent infection with high-risk HPV, mediated by viral oncoproteins E6 and E7, disrupts key cell-cycle regulators and facilitates malignant transformation.

With over 600,000 cases estimated annually, cervical cancer is the fourth-most common cancer in women globally. Although only a small proportion of HPV infections progress to high-grade CIN or invasive cancer, the global incidence of cervical cancer remains high, particularly in low- and middle-income countries [3]. In Israel, approximately 180–260 women are diagnosed annually with invasive cervical cancer and around 2000 with high-grade precancerous lesions. Most cases are diagnosed at an early or localized stage, and overall incidence and mortality are relatively low compared with many other countries, yet significant gaps remain in screening uptake and outcomes across socio-economic and ethnic groups [4].

Secondary prevention through Pap cytology and HPV DNA testing has substantially reduced cervical cancer incidence and mortality where organized screening programs exist. In Israel, current national guidelines recommend a Pap screening every three years for women aged 25–54 and Pap testing is included in the national health basket since 2021 (and for ages 30–54 prior to 2021). However, recent data show that only about half of eligible women undergo screening within the recommended interval, with lower coverage among women with lower socio-economic status and in minority populations [4].

Prophylactic HPV vaccines based on virus-like particles (VLPs) of the L1 capsid protein provide highly effective primary prevention. The currently available bivalent, quadrivalent, and 9-valent vaccines target HPV-16 and HPV-18, with the quadrivalent and 9-valent formulations also covering low-risk types 6 and 11, as well as other additional high-risk types. Clinical trials and post-licensure observational studies have demonstrated the high efficacy of HPV vaccination in preventing HPV infection, anogenital warts, and high-grade CIN among HPV-naïve adolescents and young adults. Evidence on the impact of vaccination on invasive cervical cancer, while growing, is still relatively recent and more limited [5].

In Israel, the quadrivalent HPV vaccine was introduced in 2013 into the national immunization program for eighth-grade girls, later extended to boys, and subsequently replaced by the 9-valent vaccine. However, this coverage has fluctuated and varies substantially by ethnicity and level of religious observance [6]. To date, relatively few real-world studies have evaluated the effectiveness of HPV vaccination in Israel [7], and none, to our knowledge, have focused on cervical cancer outcomes using large routine care datasets.

The primary objective of this study was to assess the relationship between HPV vaccination and the risk of cervical cancer and high-grade cervical lesions in a large cohort of Israeli women. Specifically, we aimed to generate early real-world evidence to evaluate vaccine effectiveness in preventing cervical cancer and precancerous lesions in Israel, and to explore whether the age at vaccination modifies vaccine effectiveness, with a focus on vaccination before ages 18 and 20.

## 2. Materials and Methods

### 2.1. Study Design and Data Source

We conducted a historical retrospective cohort study using data from Maccabi Healthcare Services (MHS), the second-largest health maintenance organization in Israel which serves over 2.6 million members with nationwide coverage. MHS operates fully computerized medical records that include demographic information, clinical diagnoses, procedures, laboratory results, imaging, pharmacy dispensations, and linkage to national registries.

Analyses were performed on a synthetic derivative of the MHS database. Synthetic datasets are generated to preserve the joint statistical properties of real data while protecting patient privacy and have been shown to provide valid epidemiologic inferences. This statistical process was used to elicit information from actual data and re-express it as a collection of artificial datasets for public consumption [8]. This allowed the preservation of the inferential utility of the actual dataset while preventing individual identity disclosure. We utilized MDClone (Beer Sheva, Israel), a data synthesis platform that uses computational derivation methods, to produce such de-identified, synthetic datasets based on real health systems’ data. Previous studies using MDClone have demonstrated its utility and efficiency, as the data was shown to maintain similar statistical properties compared to their real data sources [9]. The cancer registry within MHS is based on the Israel National Cancer Registry, which has mandatory reporting and high completeness.

### 2.2. Selection Criteria

The source population comprised female MHS members who underwent a first Pap smear at the age 18 or older between 1 January 2014 and 31 December 2015. The date of the first Pap test during this period was defined as the index date. We excluded women with a documented diagnosis of cervical cancer prior to this index Pap test. These criteria were chosen to reduce surveillance bias, as HPV-vaccinated women were more likely to participate in cervical cancer screening. We acknowledge that defining HPV vaccination prior to cohort entry introduces a period of immortal time. This design choice reflects a deliberate trade-off between minimizing immortal time [10] and reducing differential surveillance bias [11] by ensuring uniform baseline cervical screening at cohort entry (see Section 4.2). After applying inclusion and exclusion criteria, the analytic cohort consisted of 98,102 women. Of these, 9198 had received at least one dose of the HPV vaccine before the index date, while 88,904 were unvaccinated. For age-stratified analyses, we created sub-cohorts of women vaccinated before age 18 and before age 20.

### 2.3. HPV Vaccination

The exposure of interest was the receipt of HPV vaccination prior to the index Pap smear. Vaccination statuses and dates were obtained from MHS immunization records. Women were classified as “vaccinated” if they had a documented receipt of the HPV vaccine before the index date and as “unvaccinated” otherwise. Their age at the first HPV vaccine dose was used for subgroup analyses (<18 years and <20 years).

### 2.4. Outcomes

The primary outcome was incident cervical cancer, identified through the MHS cancer registry, which is derived from the Israel National Cancer Registry. Secondary outcomes included (1) any cervical pathology (a composite of cervical cancer, CIN1–3, and carcinoma in situ of the cervix) and (2) carcinoma in situ and invasive cervical cancer analyzed separately. Follow-ups began on the index date and continued until the earliest of: outcome diagnosis, death, disenrollment from MHS, or 31 December 2022.

### 2.5. Covariates

Potential confounders were assessed at a baseline (index date) and included: age (continuous), country of birth (Israel, Asia, Eastern Europe, and other), immigrant status, smoking status (ever vs. never), and history of sexually transmitted infections (STIs), including genital herpes, Chlamydia trachomatis, and others, as proxies for sexual risk behavior. We also collected information on history of anogenital warts and other HPV-related warts, history of any cancer, autoimmune, and immunocompromising conditions based on diagnostic codes and an aggregated MHS’s “immune deficiency” registry, and influenza vaccination history (ever vs. never) as a proxy for health literacy and preventive behavior. These covariates were chosen to capture a baseline for health status, immune function, and behaviors that may influence both the likelihood of HPV vaccination and the risk of cervical cancer.

### 2.6. Statistical Analysis

The outcome was modeled as time-to-event, with the occurrence of cervical cancer (or specific cervical endpoints) as the event of interest and censoring at death, disenrollment, or end of follow-ups. We first described baseline characteristics of vaccinated and unvaccinated women using means (standard deviations) or proportions, and compared them using t-tests, chi-square tests, or Mann–Whitney U tests where appropriate. To better account for confounding by indication, we employed propensity score weighting.

The propensity score (PS) was used to consider covariates, including demographics such as age (as a continuous variable), immigration status and country of birth, health behavior indices such as smoking status, documented influenza vaccination, and history of sexually transmitted infections, comorbidities (cancer history immunocompromised patients, and history of genital warts), co-medications (systemic corticosteroids, thyroid hormone, warfarin, and other oral diabetic drugs). The PS represented a conditional probability that each patient will be immunized with the HPV vaccine under a given covariate as a way to correct for covariates between the study groups. In this study, we calculated PS through a logistic regression model with HPV vaccination as a dependent variable and all pre-specified covariates (Supplementary Table S1). We then calculated the inverse probability of treatment weights: 1/PS for vaccinated women and 1/(1 − PS) for unvaccinated women, thereby creating a weighted pseudopopulation in which baseline covariates were balanced across the exposure groups. The balance was assessed using standardized mean differences (SMDs), with values ≤0.1 considered indicative of negligible imbalance.

Weighted Kaplan–Meier curves were constructed to compare the cumulative incidence of cervical pathology by vaccination status, and the log-rank test was used to assess differences between these groups. Weighted Cox proportional hazards models were fitted to estimate HRs and 95% CIs for the association between HPV vaccination and each outcome using the Breslow estimator [12] and a robust sandwich variance estimator. Vaccine effectiveness (VE) was expressed as (1 − HR) × 100%. Many studies used age 18–20 years as a cutoff point between different age groups, likely reflecting the average age of sexual debut. Therefore, models were run for the overall cohort and subgroups were defined within by the age at vaccination (<18 and <20 years). Proportional hazards assumptions were assessed using standard diagnostics. R program (version 4.4.2) was used for all analyses (R Foundation for Statistical Computing, Vienna, Austria).

### 2.7. Sample Size Considerations

Based on preliminary assumptions, we anticipated a cumulative incidence of cervical cancer of approximately 6% over nine years among unvaccinated women, and we hypothesized a minimum vaccine effectiveness of 20%. With α = 0.05 and 90% power, a sample of at least 7453 women per group was required. Given MHS population figures and Pap testing rates, we expected around 80,000 women with a first Pap test in 2014–2015, with roughly 10% vaccinated—adequate to meet these requirements.

### 2.8. Ethics

The study used fully synthetic, de-identified data derived from MHS databases. According to institutional and national guidelines, analyses on synthetic data that cannot be traced back to individual patients do not require Institutional Review Board or Helsinki Committee approval. The use of synthetic data ensured full protection of personal privacy while preserving the analytic validity of the findings.

## 3. Results

### 3.1. Study Population

After excluding 300 women with a history of cervical cancer before the index Pap test, the analytic cohort comprised 98,102 women. Of these, 9198 (9.4%) had received the HPV vaccination prior to this index, and 88,904 (90.6%) were unvaccinated.

Following propensity score weighting, 81,291.3 weighted counts were vaccinated and 98,281.4 were unvaccinated in the pseudopopulation. Table 1 reports the unweighted and weighted results for patient demographic and baseline clinical characteristics. Weighted baseline characteristics were well balanced between groups, with SMDs below 0.1 for most variables. The mean age was 41.7 (SD 11.0 years) and 38.1 years (SD 8.2 years) in vaccinated women, and 42.9 (SD 10.7 years) and 41.7 years (SD 11.0 years) in unvaccinated women before and after weighting, respectively. The SMD was 0.366, indicating a small-to-moderate imbalance and supporting the need for age-stratified analyses. The proportions of immigrants, women with prior cancer, STIs, anogenital warts, previous influenza vaccination, and ever-smokers were comparable between groups in the weighted sample.

### 3.2. HPV Vaccination and Risk of Cervical Pathology

During the follow-up period, there were 582 incident cases of carcinoma in situ and 60 cases of invasive cervical cancer in the relevant analytic subsets. The cumulative incidence of cervical pathology among vaccinated and unvaccinated women after 8 years of follow-ups were 2.4% and 0.7%, respectively.

In the overall weighted cohort, HPV-vaccinated women had a substantially higher risk of any cervical pathology (unweighted number of cases, 766) compared with unvaccinated women (HR 3.18, 95% CI 2.47–4.11, and *p* < 0.001; Table 2).

**Figure 1:**
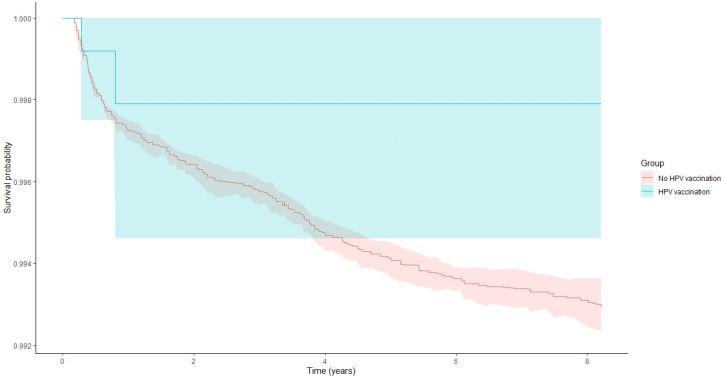
Kaplan–Meier curves displaying cervical pathology survival probability and 95% confidence intervals among study patients in women that were vaccinated with HPV vaccine prior to age 20 years and among those not vaccinated (log-rank test *p* = 0.001).

When analyses were restricted to women vaccinated before age 18, HPV vaccination was associated with a markedly reduced risk of cervical cancer, although it did not reach the pre-specified threshold for statistical significance (HR 0.28, 95% CI 0.07–1.20, and *p* = 0.0874). Similar results were observed among women vaccinated before age 20 (HR = 0.28, 95%CI: 0.04–1.98, and *p* = 0.20), again indicating a negative association that did not reach statistical significance, likely due to limited power (Figure 1).

When examining specific outcomes, HPV vaccination was associated with an increased risk of carcinoma in situ (HR 3.62, 95% CI 2.80–4.68, and *p* < 0.001), whereas the association with invasive cervical cancer (60 cases) was not statistically significant (HR 2.33, 95% CI 0.74–7.39, and *p* = 0.15).

## 4. Discussion

In this large retrospective cohort of Israeli women undergoing their first Pap testing in MHS, we observed a complex relationship between HPV vaccination and cervical cancer risk.

In the overall cohort, HPV-vaccinated women exhibited a significantly higher risk of diagnosed cervical pathology, particularly carcinoma in situ, compared with unvaccinated women. This counterintuitive finding is unlikely to reflect a true causal increase in risk from the vaccine, given extensive evidence from clinical trials and real-world studies [13,14] demonstrating vaccine safety and protective effects against HPV-related disease. Instead, it most plausibly reflects residual confounding and differential baseline risks between vaccinated and unvaccinated women, including socio-demographic, cultural, and behavioral factors not fully captured in our dataset.

By contrast, in analyses restricted to women vaccinated before age 18 or 20, we found a strong trend toward reduced cervical cancer risk, consistent with the biological rationale that vaccination was most effective when administered before HPV exposure and with international evidence from countries with long-standing vaccination programs. Although the associations in these subgroups did not reach conventional statistical significance, the magnitude and direction of the HRs are compatible with substantial vaccine effectiveness, and the lack of significance is likely due to a limited sample size and event counts in these subgroups.

### 4.1. Comparison with Previous Studies

Our findings align with multiple international studies showing that HPV vaccination in adolescence significantly reduces the incidence of high-grade CIN and invasive cervical cancer. Population-based data from Scotland and other countries have reported near elimination of cervical cancer in women vaccinated at ages 12–13, and marked reductions in disease when vaccination occurred before age 18 [15]. A recently published Cochrane meta-analysis on the impact of HPV vaccination before age 16 indicated a pooled relative risk of 0.20 (95% CI 0.09 to 0.44) for cervical cancer and 0.26 (95% CI 0.12 to 0.56) for CIN3+ [16]. Other meta-analyses of cohort and case–control studies have similarly highlighted the critical importance of age at vaccination, with the highest effectiveness observed when vaccination is completed before age 15 and diminishing effectiveness with older ages at first dose [17].

In Israel, previous work using MHS data has demonstrated a reduction in anogenital warts following the introduction of the quadrivalent vaccine [7], but to our knowledge, this is the first study to examine cervical cancer outcomes in an Israeli population using large-scale routine care data. Our observation of a protective trend among women vaccinated before age 18–20 is therefore an important early signal of real-world vaccine effectiveness in this context.

The apparent increased risk of cervical pathology among women vaccinated at older ages parallels some international findings indicating that “catch-up” vaccinations in sexually active adults offer less protection and may be selectively taken up by women already at a higher baseline risk (e.g., due to sexual behavior, high health literacy, or prior abnormal screening). In such settings, confounded by indication and surveillance bias, are substantial challenges. Although we restricted the cohort to women having a first Pap test in 2014–2015 to mitigate this, differential screening intensity during follow-ups may still exist [11].

### 4.2. Potential Sources of Bias and Confounding

In addition to potential indication and surveillance bias, there are several factors that may account for the observed increased risk in the overall vaccinated group, such as socio-cultural and religious factors. In Israel, HPV vaccine uptake is strongly associated with ethnicity and level of religious observance; Arab and secular Jewish populations tend to have higher uptake, whereas ultra-Orthodox Jewish communities have lower coverage. At the same time, sexual behavior patterns and baseline risk of HPV exposure differ markedly between these groups. Women from ultra-Orthodox communities may be less likely to be vaccinated yet also have fewer sexual partners and lower HPV exposure, thus forming a low-risk reference group compared with vaccinated women from other backgrounds [18]. Our dataset lacked direct measures of sexual behavior (e.g., age at sexual debut or number of partners) and some socio-economic indicators. We attempted to use proxies, such as STI history, anogenital warts, and influenza vaccination, to capture aspects of risk and preventive behavior; however, residual confounding is likely. The plausibility of such residual confounding has been supported by the prior literature. For example, a large study among U.S. college students found that sexually naïve individuals were less likely to initiate HPV vaccination compared with those who had previously engaged in sexual activity [19]. In this context, unmeasured differences in sexual behavior and related socio-cultural factors may well explain the higher observed rates of cervical pathology among vaccinated women compared with unvaccinated women older than 20 years.

The period between HPV vaccination and the index Pap test constitutes an “immortal” interval, as inclusion in the vaccinated group requires survival and continued membership until the beginning of the follow-up. However, we believe that the magnitude of this requirement in our study is likely limited for several reasons. First, background mortality in the relevant population is extremely low: according to Israeli life tables (2017–2021), cumulative mortality among females aged 18–40 years (the mean age at index) is approximately 0.76% [20]. Second, the annual incidence of invasive cervical cancer among women aged under the recommended screening age (35–54) is very low (0–6 per 100,000) [21], making disease-related attrition during the immortal period unlikely. Third, annual membership discontinuation rates in MHS are low (e.g., 1.6% in 2017) [22], suggesting minimal differential loss to follow-ups between vaccination and index.

### 4.3. Strengths and Limitations

The strengths of this study include the large sample size; use of comprehensive, routinely collected data from a major health provider; linkage to a high-quality national cancer registry; and a long follow-up (up to eight years) period. The focus on women at the time of their first Pap test partially helped standardize screening exposure, and the use of propensity score weighting improved the balance in observed covariates between vaccinated and unvaccinated groups.

Limitations include the observational design and associated risk of residual confounding, especially by sexual behavior and socio-cultural factors not directly measured; potential surveillance bias; and relatively low numbers of invasive cervical cancer cases, limiting power for some subgroup analyses. The reliance on synthetic data, while ethically advantageous, may introduce minor distortions, although overall associations are expected to be preserved. Finally, the follow-up time may still be insufficient to fully capture the long-term impact of HPV vaccination on invasive cervical cancer, particularly among women vaccinated at young ages.

### 4.4. Implications for Public Health and Future Research

Despite these limitations, our findings demonstrate support for early vaccination. The observed protective trend among women vaccinated before age 18–20 reinforces current recommendations to vaccinate adolescents prior to sexual debut, and underscores the importance of maintaining and improving coverage in these age groups.

Future research using larger national cohorts, longer follow-ups, and richer socio-behavioral data is warranted to confirm and refine these findings, and to further guide policy decisions regarding HPV vaccination strategies in Israel.

## 5. Conclusions

In this retrospective cohort of Israeli women, HPV vaccination in adulthood was associated with a higher observed risk of cervical pathology, likely reflecting residual confounding and surveillance biases, while vaccination in adolescence showed a strong, though not yet statistically significant, trend toward reduced cervical cancer risk. These results are consistent with international evidence that HPV vaccination is most effective when administered at younger ages, before HPV exposure.

According to a report from the Israel Ministry of Health, during 2019–2023 the uptake of the first dose of the HPV vaccination (Gardasil 9™) among eighth-grade students was 63% [23], which was lower than the average reported in high-income countries (72%) [24]. This gap has been attributed primarily to parental refusal (17%) and school-level refusal (14%). Our findings, which demonstrate high real-world effectiveness of HPV vaccination administered before age 18, underscore the importance of strengthening efforts to increase vaccine uptake and to achieve the World Health Organization’s target of 90% HPV vaccination coverage by age 15 by 2030 [25].

Our findings add to the growing body of evidence that HPV vaccination is related to greater effectiveness against cervical outcomes when administered before age 18 [26]. Continued monitoring of vaccinated cohorts, with particular attention to socio-cultural and behavioral determinants, is essential to fully assess the long-term impact of the HPV vaccination program on cervical cancer prevention in Israel.

## Figures and Tables

**Table 1:** Baseline characteristics of the unweighted and weighted pseudopopulation by HPV vaccination status.

	Raw Data (Unweighted)			Weighted Data		
	No Vaccinated for HPV	Vaccinated for HPV	SMD	No Vaccinated for HPV	Vaccinated for HPV	SMD
n	88,904	9198		98,281.4	81,291.3	
Age y, mean(SD)	42.85 (10.67)	30.49 (5.36)	1.432	41.65 (10.99)	38.11 (8.15)	0.366
Immigrant (%)	19,061 (21.4%)	1127 (12.3)	0.247	20,221(20.6)	18,524.1 (22.8)	0.054
Smoking, ever (%)	28,693 (32.3)	3077 (33.5)	0.025	31,883 (32.4)	26,072.3 (32.1)	0.008
Cancer history (%)	4375 (4.9)	129 (1.4)	0.202	44,999 (4.6)	2755.6 (3.4)	0.061
STI (%)	49 (0.1)	2 (<0.01)	0.017	51 (0.1)	17.2 (0.02)	0.016
Genital warts (%)	11,485 (12.9)	2211 (24.0)	0.289	13,796(14.0)	11,689.1 (14.4)	0.01
Flu shot, ever	34,161 (38.4)	3785 (41.2)	0.056	38,043 (38.7)	29,878.6 (36.8)	0.04

STI, sexually transmitted infections; SMD, standardized mean difference.

**Table 2:** Association between HPV vaccination and cervical outcomes in the weighted cohort (overall).

Outcome	HPV Vaccine	HR *	95% CI	*p*
Any cervical pathology	No	1 (ref)		
	Yes	3.18	2.47–4.11	<0.001
Carcinoma in situ	No	1 (ref)		
	Yes	3.62	2.80–4.68	<0.001
Invasive cancer	No	1 (ref)		
	Yes	2.33	0.74–7.39	0.15

* Models incorporated inverse probability of HPV vaccination weights. See text for details.

## Data Availability

Data is available with permission from MHS’s KSM research institute.

## Supplementary Materials

The following supporting information can be downloaded at: https://www.mdpi.com/article/10.3390/jcm15030995/s1, Table S1: Logistic regression coefficients for propensity scores to predict HPV vaccination status [27].

## Abbreviations

The following abbreviations are used in this manuscript:

HPV	Human papillomavirus
MHS	Maccabi Healthcare Services
HR	Hazards ratio
CI	Confidence interval
CIN	Cervical intraepithelial neoplasia
PS	Propensity score

## References

[1] Meng X., Yang B., Yin H., Chen J., Ma W., Xu Z., Shen Y. (2025). Global Burden and Incidence Trends in Cancers Associated with Human Papillomavirus Infection: A Population-Based Systematic Study. Pathogens.

[2] Jensen J.E., Becker G.L., Jackson J.B., Rysavy M.B. (2024). Human papillomavirus and associated cancers: A review. Viruses.

[3] Singh D., Vignat J., Lorenzoni V., Eslahi M., Ginsburg O., Lauby-Secretan B., Arbyn M., Basu P., Bray F., Vaccarella S. (2023). Global estimates of incidence and mortality of cervical cancer in 2020: A baseline analysis of the WHO Global Cervical Cancer Elimination Initiative. Lancet Glob. Health.

[4] Israel Center for Disease Control Cervical Cancer in Israel—Data Update, January 2023. Jerusalem: Israel Ministry of Health; 2025. https://www.gov.il/en/pages/cervical-cancer-2023.

[5] Wang R., Pan W., Jin L., Huang W., Li Y., Wu D., Gao C., Ma D., Liao S. (2020). Human papillomavirus vaccine against cervical cancer: Opportunity and challenge. Cancer Lett..

[6] Wortsman J., Glaser Chodik N., Chodick G. (2023). Correlations of HPV vaccine uptake among eight-grade students in Israel: The importance of ethnicity and level of religious observance. Women Health.

[7] Lurie S., Mizrachi Y., Chodick G., Katz R., Schejter E. (2017). Impact of quadrivalent human papillomavirus vaccine on genital warts in an opportunistic vaccination structure. Gynecol. Oncol..

[8] Raghunathan T. (2021). Synthetic Data. Annu. Rev. Stat. Appl..

[9] Wang E., Mott K., Zhang H., Gazit S., Chodick G., Burcu M. (2024). Validation Assessment of Privacy-Preserving Synthetic Electronic Health Record Data: Comparison of Original Versus Synthetic Data on Real-World COVID-19 Vaccine Effectiveness. Pharmacoepidemiol. Drug Saf..

[10] Platt R.W., Hutcheon J.A., Suissa S. (2019). Immortal Time Bias in Epidemiology. Curr. Epidemiol. Reports.

[11] Chodick G., Leader A.E., Larson S. (2021). Catch-up HPV Vaccination and Subsequent Uptake of Papanicolaou Testing in A State-mandated Health System. Cancer Prev. Res..

[12] Breslow N.E. (1975). Analysis of survival data under the proportional hazards model. Int. Stat. Rev. Rev. Int. Stat..

[13] Lin R., Jin H., Fu X. (2023). Comparative efficacy of human papillomavirus vaccines: Systematic review and network meta-analysis. Expert. Rev. Vaccines.

[14] Kurosawa M., Sekine M., Yamaguchi M., Kudo R., Hanley S.J.B., Hara M., Adachi S., Ueda Y., Miyagi E., Ikeda S. (2022). Long-Term Effects of Human Papillomavirus Vaccination in Clinical Trials and Real-World Data: A Systematic Review. Vaccines.

[15] Palmer T.J., Kavanagh K., Cuschieri K., Cameron R., Graham C., Wilson A., Roy K. (2024). Invasive cervical cancer incidence following bivalent human papillomavirus vaccination: A population-based observational study of age at immunization, dose, and deprivation. JNCI J. Natl. Cancer Inst..

[16] Henschke N., Bergman H., Buckley B.S., Crosbie E.J., Dwan K., Golder S.P., Kyrgiou M., Loke Y.K., McIntosh H.M., Probyn K. (2025). Effects of human papillomavirus (HPV) vaccination programmes on community rates of HPV-related disease and harms from vaccination. Cochrane Database Syst. Rev..

[17] Ellingson M.K., Sheikha H., Nyhan K., Oliveira C.R., Niccolai L.M. (2023). Human papillomavirus vaccine effectiveness by age at vaccination: A systematic review. Hum. Vaccin. Immunother..

[18] Shahbari N.A.E., Gesser-Edelsburg A., Davidovitch N., Brammli-Greenberg S., Grifat R., Mesch G.S. (2021). Factors associated with seasonal influenza and HPV vaccination uptake among different ethnic groups in Arab and Jewish society in Israel. Int. J. Equity Health.

[19] Adjei Boakye E., McKinney S.L., Whittington K.D., Boyer V.E., Franca M.C., Lee M., McKinnies R.C., Collins S.K., Gerend M.A. (2022). Association between Sexual Activity and Human Papillomavirus (HPV) Vaccine Initiation and Completion among College Students. Vaccines.

[20] Israel Bureau of Statistics (2023). Complete Life Tables, Israel 2017–2021. https://www.cbs.gov.il/he/publications/DocLib/2023/1911_life_tables_2017_2021/e_print.pdf.

[21] Israel Ministry of Health National Cancer Registry data. https://public.tableau.com/app/profile/mohbi/viz/_17315087730200/sheet4.

[22] Israel National Security Membership in Sick Funds, 2017. https://www.btl.gov.il/Publications/survey/Documents/seker_303.pdf.

[23] Israel Ministry of Health Public-Health-Services-Summary-Report-2022-2023. https://www.gov.il/BlobFolder/reports/public-health-services-summary-report-2022-2023/he/files_publications_units_public_health_services_Public-Health-services-summary-report-2022-2023.pdf.

[24] Han J., Zhang L., Chen Y., Zhang Y., Wang L., Cai R., Li M., Dai Y., Dang L., Chen H. (2025). Global HPV vaccination programs and coverage rates: A systematic review. EClinicalMedicine.

[25] World Health Organization (2020). Global Strategy to Accelerate the Elimination of Cervical Cancer as a Public Health Problem.

[26] Oliveira C.R., Shapiro E.D., Sheth S.S., Ellingson M.K., Johnson N.P., Sullivan E.L., Querec T.D., Unger E.R., Niccolai L.M. (2025). Clinical effectiveness of HPV vaccine by age at vaccination: A matched case-control study. Lancet Reg Health Am..

[27] Shapiro Ben David S., Goren I., Mourad V., Cahan A. (2022). Vaccination coverage among immunocompromised patients in a large health maintenance organization: Findings from a novel computerized registry. Vaccines.

