# Supplementary material for: Association Between Human Papillomavirus Vaccination and the Risk of Cervical Cancer and Precancerous Lesions in Israel: A Retrospective Cohort Study
Source: J Clin Med. 2026 Jan 26;15(3):995. doi: 10.3390/jcm15030995 (PMC12898192; doi:10.3390/jcm15030995)
Supplement: Supplementary file 1 [file jcm-15-00995-s001.zip › jcm-4076127-supplementary.pdf]

Table S1. Logistic regression coefficients for propensity scores to predict HPV vaccination status

Variable	Coefficient	Standard error	OR	95% CI	p-value
Age (per year)	-0.17	0.002	0.84	0.84 – 0.85	<0.001
Immigrant (yes vs. no)	-0.49	0.03	0.61	0.57 – 0.66	<0.001
Smoking status (ever vs. never)	0.20	0.03	1.22	1.16 – 1.29	<0.001
Immunocompromised individuals(1) (yes vs. no)	0.07	0.06	1.08	0.96 – 1.22	0.21
History of genital warts (yes vs. no)	0.63	0.03	1.89	1.78 – 2.00	<0.001
Cancer history (yes vs. no)	0.01	0.10	1.01	0.83 – 1.22	0.92
History of sexually transmitted infections (yes vs. no)	-0.09	0.75	0.91	0.21 – 3.96	0.91
Influenza vaccination (yes vs. no)	0.55	0.03	1.73	1.65 – 1.82	<0.001

1. Shapiro Ben David S, Goren I, Mourad V, Cahan A. Vaccination coverage among immunocompromised patients in a large health maintenance organization: findings from a novel computerized registry. Vaccines. 2022;10(10):1654.
